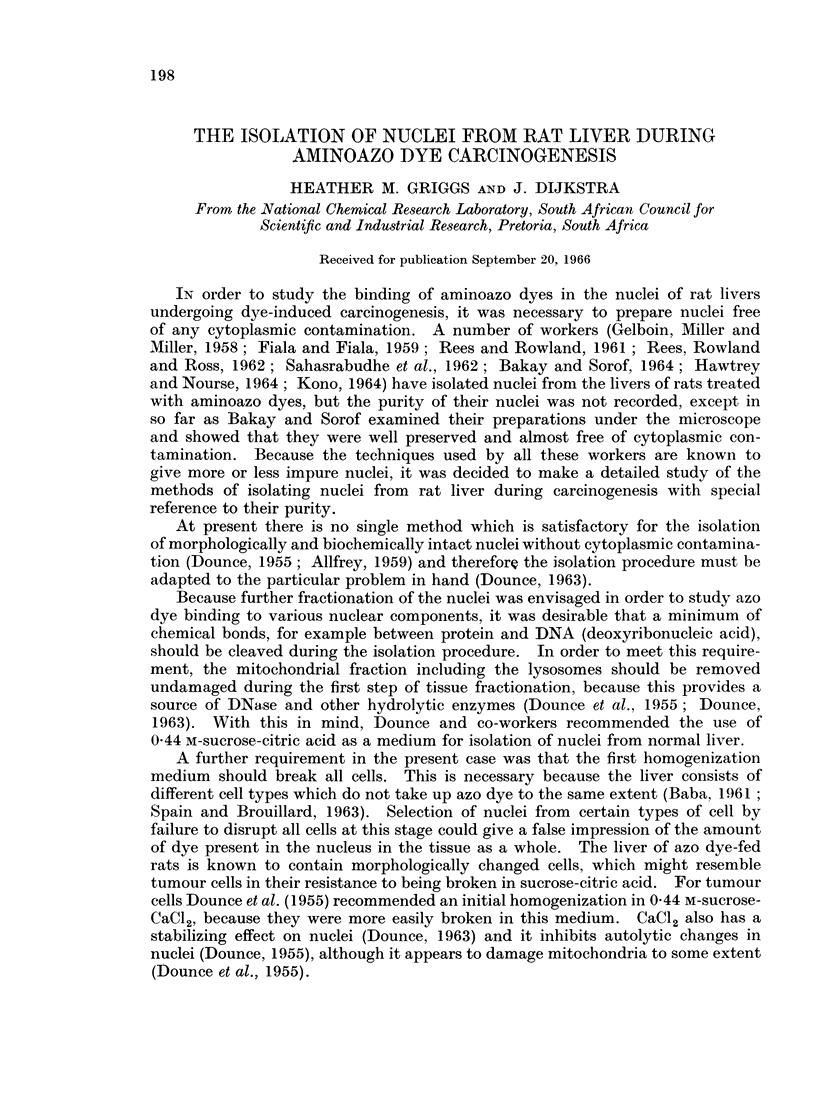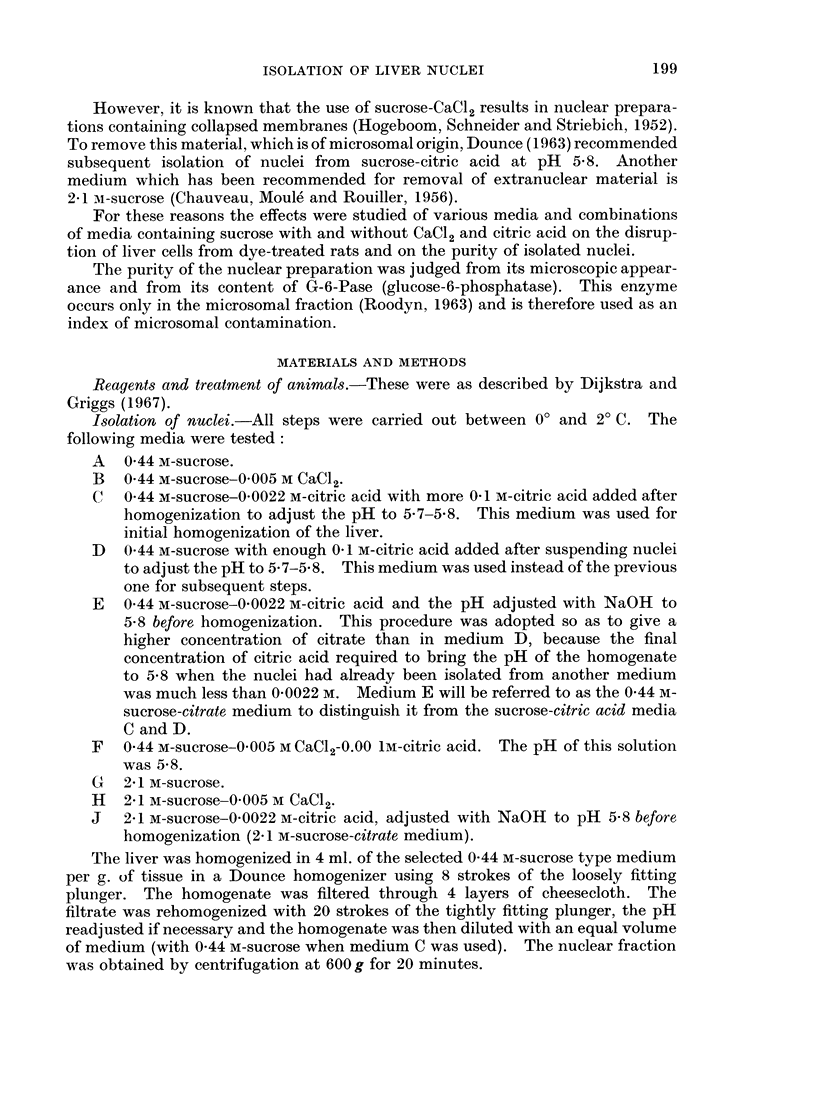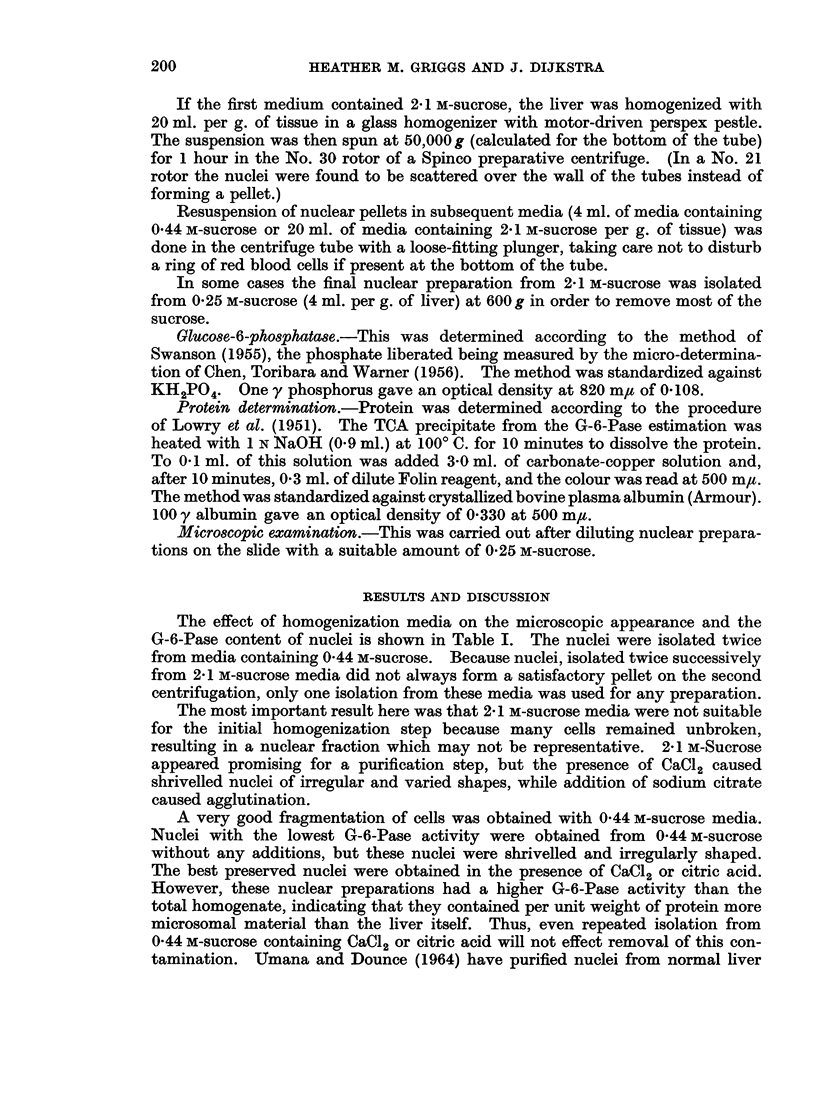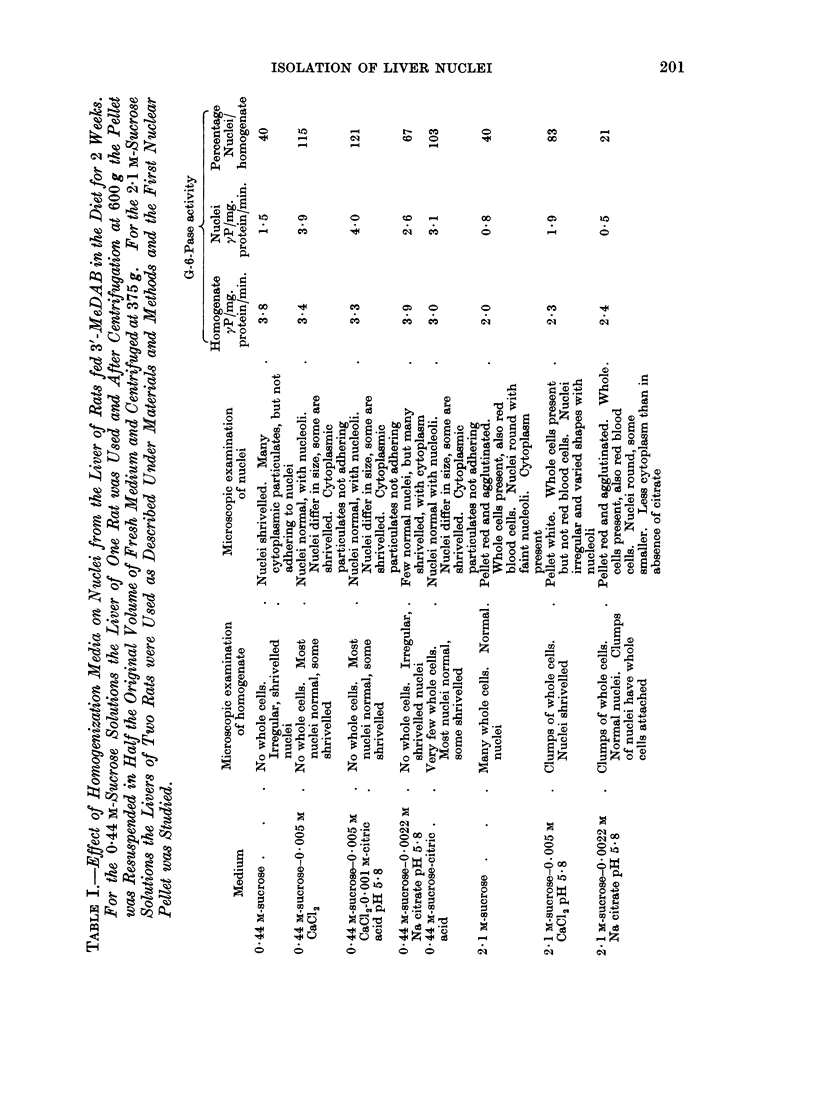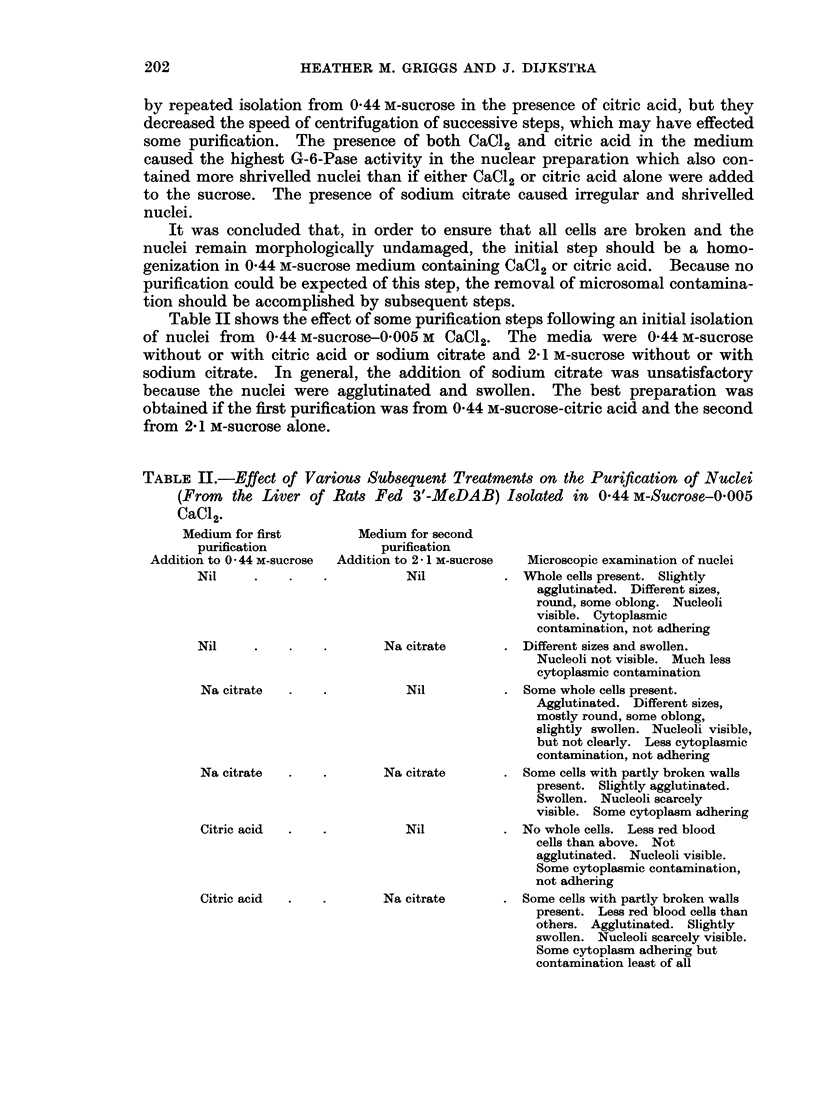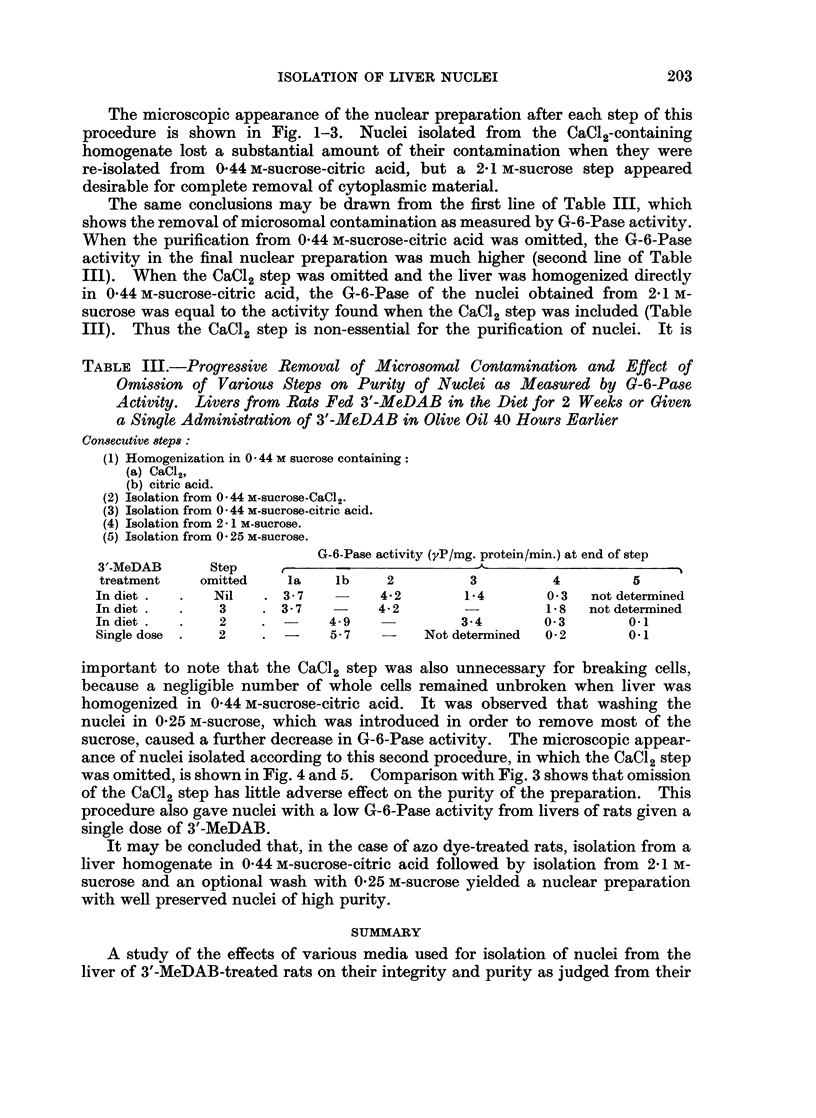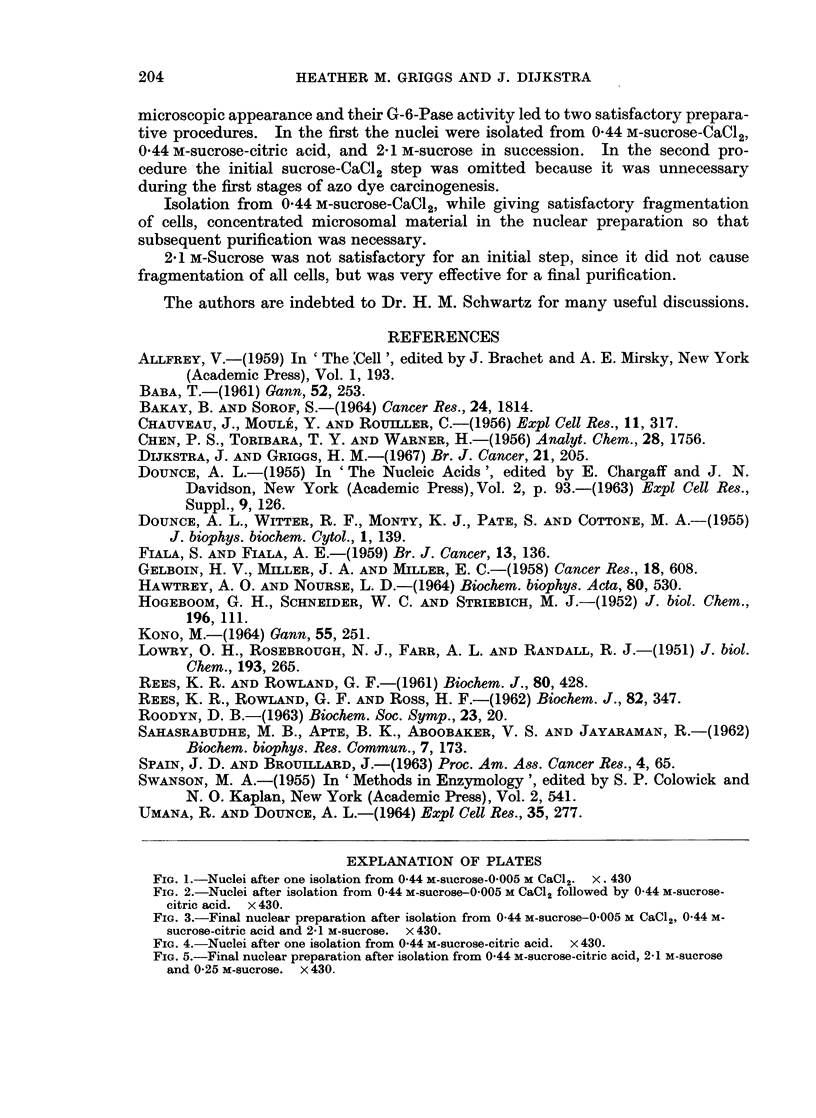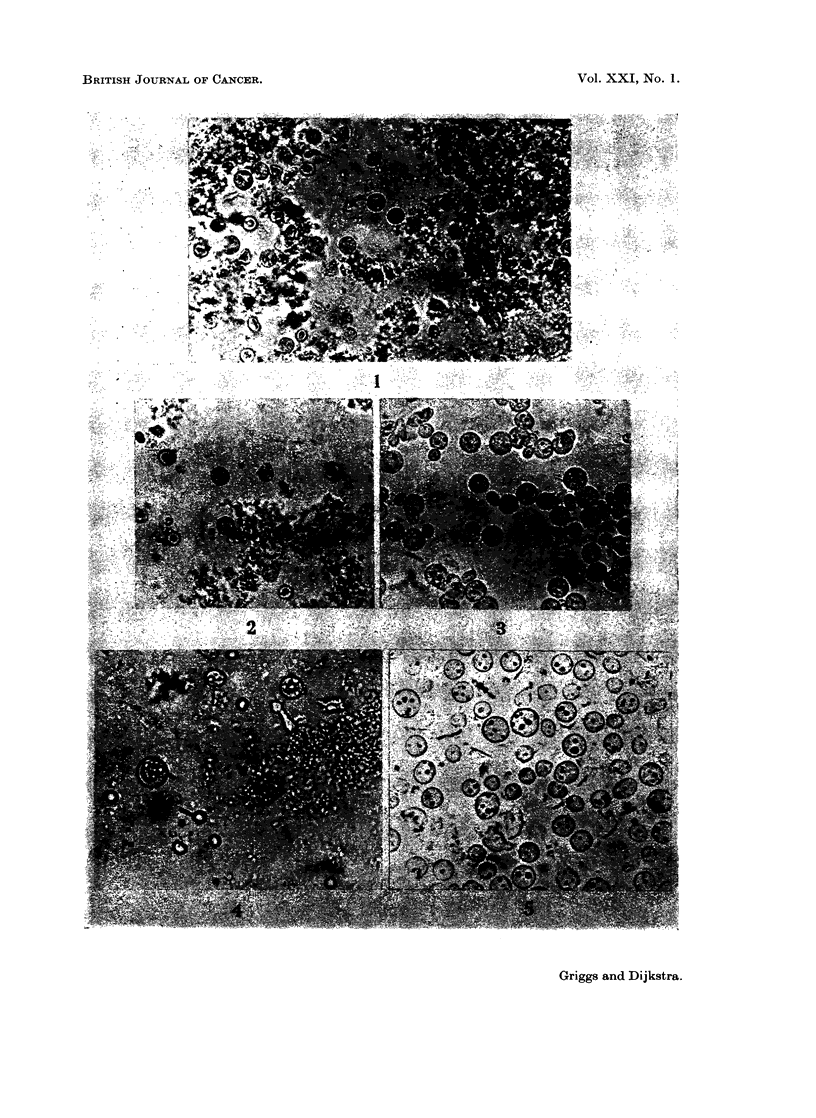# The isolation of nuclei from rat liver during aminoazo dye carcinogenesis.

**DOI:** 10.1038/bjc.1967.21

**Published:** 1967-03

**Authors:** H. M. Griggs, J. Dijkstra

## Abstract

**Images:**


### THE ISOLATION OF NUCLEI FROM RAT LIVER DURING AMINOAZO DYE CARCINOGENESIS

HEATHER M. GRIGGS AND J. DIJKSTRA

*From the National Chemical Research Laboratory, South African Council for Scientific and Industrial Research, Pretoria, South Africa*

Received for publication September 20, 1966

IN order to study the binding of aminoazo dyes in the nuclei of rat livers undergoing dye-induced carcinogenesis, it was necessary to prepare nuclei free of any cytoplasmic contamination. A number of workers (Gelboin, Miller and Miller, 1958 ; Fiala and Fiala, 1959 ; Rees and Rowland, 1961 ; Rees, Rowland and Ross, 1962 ; Sahasrabudhe *et al.*, 1962 ; Bakay and Sorof, 1964 ; Hawtrey and Nourse, 1964 ; Kono, 1964) have isolated nuclei from the livers of rats treated with aminoazo dyes, but the purity of their nuclei was not recorded, except in so far as Bakay and Sorof examined their preparations under the microscope and showed that they were well preserved and almost free of cytoplasmic contamination. Because the techniques used by all these workers are known to give more or less impure nuclei, it was decided to make a detailed study of the methods of isolating nuclei from rat liver during carcinogenesis with special reference to their purity.

At present there is no single method which is satisfactory for the isolation of morphologically and biochemically intact nuclei without cytoplasmic contamination (Dounce, 1955 ; Allfrey, 1959) and therefore the isolation procedure must be adapted to the particular problem in hand (Dounce, 1963).

Because further fractionation of the nuclei was envisaged in order to study azo dye binding to various nuclear components, it was desirable that a minimum of chemical bonds, for example between protein and DNA (deoxyribonucleic acid), should be cleaved during the isolation procedure. In order to meet this requirement, the mitochondrial fraction including the lysosomes should be removed undamaged during the first step of tissue fractionation, because this provides a source of DNase and other hydrolytic enzymes (Dounce *et al.*, 1955 ; Dounce, 1963). With this in mind, Dounce and co-workers recommended the use of 0·44 M-sucrose-citric acid as a medium for isolation of nuclei from normal liver.

A further requirement in the present case was that the first homogenization medium should break all cells. This is necessary because the liver consists of different cell types which do not take up azo dye to the same extent (Baba, 1961 ; Spain and Brouillard, 1963). Selection of nuclei from certain types of cell by failure to disrupt all cells at this stage could give a false impression of the amount of dye present in the nucleus in the tissue as a whole. The liver of azo dye-fed rats is known to contain morphologically changed cells, which might resemble tumour cells in their resistance to being broken in sucrose-citric acid. For tumour cells Dounce *et al.* (1955) recommended an initial homogenization in 0·44 M-sucrose-$CaCl_2$, because they were more easily broken in this medium. $CaCl_2$ also has a stabilizing effect on nuclei (Dounce, 1963) and it inhibits autolytic changes in nuclei (Dounce, 1955), although it appears to damage mitochondria to some extent (Dounce *et al.*, 1955).

However, it is known that the use of sucrose-$CaCl_2$ results in nuclear preparations containing collapsed membranes (Hogeboom, Schneider and Striebich, 1952). To remove this material, which is of microsomal origin, Dounce (1963) recommended subsequent isolation of nuclei from sucrose-citric acid at pH 5·8. Another medium which has been recommended for removal of extranuclear material is 2·1 M-sucrose (Chauveau, Moulé and Rouiller, 1956).

For these reasons the effects were studied of various media and combinations of media containing sucrose with and without $CaCl_2$ and citric acid on the disruption of liver cells from dye-treated rats and on the purity of isolated nuclei.

The purity of the nuclear preparation was judged from its microscopic appearance and from its content of G-6-Pase (glucose-6-phosphatase). This enzyme occurs only in the microsomal fraction (Roodyn, 1963) and is therefore used as an index of microsomal contamination.

### MATERIALS AND METHODS

*Reagents and treatment of animals.*—These were as described by Dijkstra and Griggs (1967).

*Isolation of nuclei.*—All steps were carried out between 0° and 2° C. The following media were tested:

A 0·44 M-sucrose.

B 0·44 M-sucrose–0·005 M $CaCl_2$.

C 0·44 M-sucrose–0·0022 M-citric acid with more 0·1 M-citric acid added after homogenization to adjust the pH to 5·7–5·8. This medium was used for initial homogenization of the liver.

D 0·44 M-sucrose with enough 0·1 M-citric acid added after suspending nuclei to adjust the pH to 5·7–5·8. This medium was used instead of the previous one for subsequent steps.

E 0·44 M-sucrose–0·0022 M-citric acid and the pH adjusted with NaOH to 5·8 *before* homogenization. This procedure was adopted so as to give a higher concentration of citrate than in medium D, because the final concentration of citric acid required to bring the pH of the homogenate to 5·8 when the nuclei had already been isolated from another medium was much less than 0·0022 M. Medium E will be referred to as the 0·44 M-sucrose-*citrate* medium to distinguish it from the sucrose-*citric acid* media C and D.

F 0·44 M-sucrose–0·005 M $CaCl_2$-0.00 1M-citric acid. The pH of this solution was 5·8.

G 2·1 M-sucrose.

H 2·1 M-sucrose–0·005 M $CaCl_2$.

J 2·1 M-sucrose–0·0022 M-citric acid, adjusted with NaOH to pH 5·8 *before* homogenization (2·1 M-sucrose-*citrate* medium).

The liver was homogenized in 4 ml. of the selected 0·44 M-sucrose type medium per g. of tissue in a Dounce homogenizer using 8 strokes of the loosely fitting plunger. The homogenate was filtered through 4 layers of cheesecloth. The filtrate was rehomogenized with 20 strokes of the tightly fitting plunger, the pH readjusted if necessary and the homogenate was then diluted with an equal volume of medium (with 0·44 M-sucrose when medium C was used). The nuclear fraction was obtained by centrifugation at 600 *g* for 20 minutes.

If the first medium contained 2·1 M-sucrose, the liver was homogenized with 20 ml. per g. of tissue in a glass homogenizer with motor-driven perspex pestle. The suspension was then spun at 50,000 *g* (calculated for the bottom of the tube) for 1 hour in the No. 30 rotor of a Spinco preparative centrifuge. (In a No. 21 rotor the nuclei were found to be scattered over the wall of the tubes instead of forming a pellet.)

Resuspension of nuclear pellets in subsequent media (4 ml. of media containing 0·44 M-sucrose or 20 ml. of media containing 2·1 M-sucrose per g. of tissue) was done in the centrifuge tube with a loose-fitting plunger, taking care not to disturb a ring of red blood cells if present at the bottom of the tube.

In some cases the final nuclear preparation from 2·1 M-sucrose was isolated from 0·25 M-sucrose (4 ml. per g. of liver) at 600 *g* in order to remove most of the sucrose.

*Glucose-6-phosphatase.*—This was determined according to the method of Swanson (1955), the phosphate liberated being measured by the micro-determination of Chen, Toribara and Warner (1956). The method was standardized against $KH_2PO_4$. One $\gamma$ phosphorus gave an optical density at 820 m$\mu$ of 0·108.

*Protein determination.*—Protein was determined according to the procedure of Lowry *et al.* (1951). The TCA precipitate from the G-6-Pase estimation was heated with 1 N NaOH (0·9 ml.) at 100° C. for 10 minutes to dissolve the protein. To 0·1 ml. of this solution was added 3·0 ml. of carbonate-copper solution and, after 10 minutes, 0·3 ml. of dilute Folin reagent, and the colour was read at 500 m$\mu$. The method was standardized against crystallized bovine plasma albumin (Armour). 100 $\gamma$ albumin gave an optical density of 0·330 at 500 m$\mu$.

*Microscopic examination.*—This was carried out after diluting nuclear preparations on the slide with a suitable amount of 0·25 M-sucrose.

### RESULTS AND DISCUSSION

The effect of homogenization media on the microscopic appearance and the G-6-Pase content of nuclei is shown in Table I. The nuclei were isolated twice from media containing 0·44 M-sucrose. Because nuclei, isolated twice successively from 2·1 M-sucrose media did not always form a satisfactory pellet on the second centrifugation, only one isolation from these media was used for any preparation.

The most important result here was that 2·1 M-sucrose media were not suitable for the initial homogenization step because many cells remained unbroken, resulting in a nuclear fraction which may not be representative. 2·1 M-Sucrose appeared promising for a purification step, but the presence of $CaCl_2$ caused shrivelled nuclei of irregular and varied shapes, while addition of sodium citrate caused agglutination.

A very good fragmentation of cells was obtained with 0·44 M-sucrose media. Nuclei with the lowest G-6-Pase activity were obtained from 0·44 M-sucrose without any additions, but these nuclei were shrivelled and irregularly shaped. The best preserved nuclei were obtained in the presence of $CaCl_2$ or citric acid. However, these nuclear preparations had a higher G-6-Pase activity than the total homogenate, indicating that they contained per unit weight of protein more microsomal material than the liver itself. Thus, even repeated isolation from 0·44 M-sucrose containing $CaCl_2$ or citric acid will not effect removal of this contamination. Umana and Dounce (1964) have purified nuclei from normal liver

TABLE I.—*Effect of Homogenization Media on Nuclei from the Liver of Rats fed 3'-MeDAB in the Diet for 2 Weeks. For the* 0·44 M-*Sucrose Solutions the Liver of One Rat was Used and After Centrifugation at* 600 **g** *the Pellet was Resuspended in Half the Original Volume of Fresh Medium and Centrifuged at* 375 **g**. *For the* 2·1 M-*Sucrose Solutions the Livers of Two Rats were Used as Described Under Materials and Methods and the First Nuclear Pellet was Studied.*

| Medium | Microscopic examination of homogenate | Microscopic examination of nuclei | G-6-Pase activity: Homogenate γP/mg. protein/min. | G-6-Pase activity: Nuclei γP/mg. protein/min. | G-6-Pase activity: Percentage Nuclei/homogenate |
|---|---|---|---|---|---|
| 0·44 M-sucrose | No whole cells. Irregular, shrivelled nuclei | Nuclei shrivelled. Many cytoplasmic particulates, but not adhering to nuclei | 3·8 | 1·5 | 40 |
| 0·44 M-sucrose–0·005 M $CaCl_2$ | No whole cells. Most nuclei normal, some shrivelled | Nuclei normal, with nucleoli. Nuclei differ in size, some are shrivelled. Cytoplasmic particulates not adhering | 3·4 | 3·9 | 115 |
| 0·44 M-sucrose–0·005 M $CaCl_2$-0·001 M-citric acid pH 5·8 | No whole cells. Most nuclei normal, some shrivelled | Nuclei normal, with nucleoli. Nuclei differ in size, some are shrivelled. Cytoplasmic particulates not adhering | 3·3 | 4·0 | 121 |
| 0·44 M-sucrose–0·0022 M Na citrate pH 5·8 | No whole cells. Irregular, shrivelled nuclei | Few normal nuclei, but many shrivelled, with cytoplasm | 3·9 | 2·6 | 67 |
| 0·44 M-sucrose-citric acid | Very few whole cells. Most nuclei normal, some shrivelled | Nuclei normal with nucleoli. Nuclei differ in size, some are shrivelled. Cytoplasmic particulates not adhering | 3·0 | 3·1 | 103 |
| 2·1 M-sucrose | Many whole cells. Normal nuclei | Pellet red and agglutinated. Whole cells present, also red blood cells. Nuclei round with faint nucleoli. Cytoplasm present | 2·0 | 0·8 | 40 |
| 2·1 M-sucrose–0.005 M $CaCl_2$ pH 5·8 | Clumps of whole cells. Nuclei shrivelled | Pellet white. Whole cells present but not red blood cells. Nuclei irregular and varied shapes with nucleoli | 2·3 | 1·9 | 83 |
| 2·1 M-sucrose–0·0022 M Na citrate pH 5·8 | Clumps of whole cells. Normal nuclei. Clumps of nuclei have whole cells attached | Pellet red and agglutinated. Whole cells present, also red blood cells. Nuclei round, some smaller. Less cytoplasm than in absence of citrate | 2·4 | 0·5 | 21 |

by repeated isolation from 0·44 M-sucrose in the presence of citric acid, but they decreased the speed of centrifugation of successive steps, which may have effected some purification. The presence of both $CaCl_2$ and citric acid in the medium caused the highest G-6-Pase activity in the nuclear preparation which also contained more shrivelled nuclei than if either $CaCl_2$ or citric acid alone were added to the sucrose. The presence of sodium citrate caused irregular and shrivelled nuclei.

It was concluded that, in order to ensure that all cells are broken and the nuclei remain morphologically undamaged, the initial step should be a homogenization in 0·44 M-sucrose medium containing $CaCl_2$ or citric acid. Because no purification could be expected of this step, the removal of microsomal contamination should be accomplished by subsequent steps.

Table II shows the effect of some purification steps following an initial isolation of nuclei from 0·44 M-sucrose–0·005 M $CaCl_2$. The media were 0·44 M-sucrose without or with citric acid or sodium citrate and 2·1 M-sucrose without or with sodium citrate. In general, the addition of sodium citrate was unsatisfactory because the nuclei were agglutinated and swollen. The best preparation was obtained if the first purification was from 0·44 M-sucrose-citric acid and the second from 2·1 M-sucrose alone.

TABLE II.—*Effect of Various Subsequent Treatments on the Purification of Nuclei (From the Liver of Rats Fed 3′-MeDAB) Isolated in* 0·44 M-*Sucrose*–0·005 $CaCl_2$.

| Medium for first purification Addition to 0·44 M-sucrose | Medium for second purification Addition to 2·1 M-sucrose | Microscopic examination of nuclei |
|---|---|---|
| Nil | Nil | Whole cells present. Slightly agglutinated. Different sizes, round, some oblong. Nucleoli visible. Cytoplasmic contamination, not adhering |
| Nil | Na citrate | Different sizes and swollen. Nucleoli not visible. Much less cytoplasmic contamination |
| Na citrate | Nil | Some whole cells present. Agglutinated. Different sizes, mostly round, some oblong, slightly swollen. Nucleoli visible, but not clearly. Less cytoplasmic contamination, not adhering |
| Na citrate | Na citrate | Some cells with partly broken walls present. Slightly agglutinated. Swollen. Nucleoli scarcely visible. Some cytoplasm adhering |
| Citric acid | Nil | No whole cells. Less red blood cells than above. Not agglutinated. Nucleoli visible. Some cytoplasmic contamination, not adhering |
| Citric acid | Na citrate | Some cells with partly broken walls present. Less red blood cells than others. Agglutinated. Slightly swollen. Nucleoli scarcely visible. Some cytoplasm adhering but contamination least of all |

The microscopic appearance of the nuclear preparation after each step of this procedure is shown in Fig. 1–3. Nuclei isolated from the $CaCl_2$-containing homogenate lost a substantial amount of their contamination when they were re-isolated from 0·44 M-sucrose-citric acid, but a 2·1 M-sucrose step appeared desirable for complete removal of cytoplasmic material.

The same conclusions may be drawn from the first line of Table III, which shows the removal of microsomal contamination as measured by G-6-Pase activity. When the purification from 0·44 M-sucrose-citric acid was omitted, the G-6-Pase activity in the final nuclear preparation was much higher (second line of Table III). When the $CaCl_2$ step was omitted and the liver was homogenized directly in 0·44 M-sucrose-citric acid, the G-6-Pase of the nuclei obtained from 2·1 M-sucrose was equal to the activity found when the $CaCl_2$ step was included (Table III). Thus the $CaCl_2$ step is non-essential for the purification of nuclei. It is important to note that the $CaCl_2$ step was also unnecessary for breaking cells, because a negligible number of whole cells remained unbroken when liver was homogenized in 0·44 M-sucrose-citric acid. It was observed that washing the nuclei in 0·25 M-sucrose, which was introduced in order to remove most of the sucrose, caused a further decrease in G-6-Pase activity. The microscopic appearance of nuclei isolated according to this second procedure, in which the $CaCl_2$ step was omitted, is shown in Fig. 4 and 5. Comparison with Fig. 3 shows that omission of the $CaCl_2$ step has little adverse effect on the purity of the preparation. This procedure also gave nuclei with a low G-6-Pase activity from livers of rats given a single dose of 3′-MeDAB.

TABLE III.—*Progressive Removal of Microsomal Contamination and Effect of Omission of Various Steps on Purity of Nuclei as Measured by G-6-Pase Activity. Livers from Rats Fed 3′-MeDAB in the Diet for 2 Weeks or Given a Single Administration of 3′-MeDAB in Olive Oil 40 Hours Earlier*

*Consecutive steps :*

(1) Homogenization in 0·44 M sucrose containing :
 (a) $CaCl_2$,
 (b) citric acid.
(2) Isolation from 0·44 M-sucrose-$CaCl_2$.
(3) Isolation from 0·44 M-sucrose-citric acid.
(4) Isolation from 2·1 M-sucrose.
(5) Isolation from 0·25 M-sucrose.

| 3′-MeDAB treatment | Step omitted | G-6-Pase activity ($\gamma$P/mg. protein/min.) at end of step 1a | 1b | 2 | 3 | 4 | 5 |
|---|---|---|---|---|---|---|---|
| In diet | Nil | 3·7 | — | 4·2 | 1·4 | 0·3 | not determined |
| In diet | 3 | 3·7 | — | 4·2 | — | 1·8 | not determined |
| In diet | 2 | — | 4·9 | — | 3·4 | 0·3 | 0·1 |
| Single dose | 2 | — | 5·7 | — | Not determined | 0·2 | 0·1 |

It may be concluded that, in the case of azo dye-treated rats, isolation from a liver homogenate in 0·44 M-sucrose-citric acid followed by isolation from 2·1 M-sucrose and an optional wash with 0·25 M-sucrose yielded a nuclear preparation with well preserved nuclei of high purity.

### SUMMARY

A study of the effects of various media used for isolation of nuclei from the liver of 3′-MeDAB-treated rats on their integrity and purity as judged from their microscopic appearance and their G-6-Pase activity led to two satisfactory preparative procedures. In the first the nuclei were isolated from 0·44 M-sucrose-$CaCl_2$, 0·44 M-sucrose-citric acid, and 2·1 M-sucrose in succession. In the second procedure the initial sucrose-$CaCl_2$ step was omitted because it was unnecessary during the first stages of azo dye carcinogenesis.

Isolation from 0·44 M-sucrose-$CaCl_2$, while giving satisfactory fragmentation of cells, concentrated microsomal material in the nuclear preparation so that subsequent purification was necessary.

2·1 M-Sucrose was not satisfactory for an initial step, since it did not cause fragmentation of all cells, but was very effective for a final purification.

The authors are indebted to Dr. H. M. Schwartz for many useful discussions.

### REFERENCES

ALLFREY, V.—(1959) In ' The Cell ', edited by J. Brachet and A. E. Mirsky, New York (Academic Press), Vol. 1, 193.
BABA, T.—(1961) *Gann*, **52**, 253.
BAKAY, B. AND SOROF, S.—(1964) *Cancer Res.*, **24**, 1814.
CHAUVEAU, J., MOULÉ, Y. AND ROUILLER, C.—(1956) *Expl Cell Res.*, **11**, 317.
CHEN, P. S., TORIBARA, T. Y. AND WARNER, H.—(1956) *Analyt. Chem.*, **28**, 1756.
DIJKSTRA, J. AND GRIGGS, H. M.—(1967) *Br. J. Cancer*, **21**, 205.
DOUNCE, A. L.—(1955) In ' The Nucleic Acids ', edited by E. Chargaff and J. N. Davidson, New York (Academic Press), Vol. 2, p. 93.—(1963) *Expl Cell Res.*, Suppl., **9**, 126.
DOUNCE, A. L., WITTER, R. F., MONTY, K. J., PATE, S. AND COTTONE, M. A.—(1955) *J. biophys. biochem. Cytol.*, **1**, 139.
FIALA, S. AND FIALA, A. E.—(1959) *Br. J. Cancer*, **13**, 136.
GELBOIN, H. V., MILLER, J. A. AND MILLER, E. C.—(1958) *Cancer Res.*, **18**, 608.
HAWTREY, A. O. AND NOURSE, L. D.—(1964) *Biochem. biophys. Acta*, **80**, 530.
HOGEBOOM, G. H., SCHNEIDER, W. C. AND STRIEBICH, M. J.—(1952) *J. biol. Chem.*, **196**, 111.
KONO, M.—(1964) *Gann*, **55**, 251.
LOWRY, O. H., ROSEBROUGH, N. J., FARR, A. L. AND RANDALL, R. J.—(1951) *J. biol. Chem.*, **193**, 265.
REES, K. R. AND ROWLAND, G. F.—(1961) *Biochem. J.*, **80**, 428.
REES, K. R., ROWLAND, G. F. AND ROSS, H. F.—(1962) *Biochem. J.*, **82**, 347.
ROODYN, D. B.—(1963) *Biochem. Soc. Symp.*, **23**, 20.
SAHASRABUDHE, M. B., APTE, B. K., ABOOBAKER, V. S. AND JAYARAMAN, R.—(1962) *Biochem. biophys. Res. Commun.*, **7**, 173.
SPAIN, J. D. AND BROUILLARD, J.—(1963) *Proc. Am. Ass. Cancer Res.*, **4**, 65.
SWANSON, M. A.—(1955) In ' Methods in Enzymology ', edited by S. P. Colowick and N. O. Kaplan, New York (Academic Press), Vol. 2, 541.
UMANA, R. AND DOUNCE, A. L.—(1964) *Expl Cell Res.*, **35**, 277.

### EXPLANATION OF PLATES

FIG. 1.—Nuclei after one isolation from 0·44 M-sucrose-0·005 M $CaCl_2$. ×. 430

FIG. 2.—Nuclei after isolation from 0·44 M-sucrose–0·005 M $CaCl_2$ followed by 0·44 M-sucrose-citric acid. ×430.

FIG. 3.—Final nuclear preparation after isolation from 0·44 M-sucrose–0·005 M $CaCl_2$, 0·44 M-sucrose-citric acid and 2·1 M-sucrose. ×430.

FIG. 4.—Nuclei after one isolation from 0·44 M-sucrose-citric acid. ×430.

FIG. 5.—Final nuclear preparation after isolation from 0·44 M-sucrose-citric acid, 2·1 M-sucrose and 0·25 M-sucrose. ×430.